# Salicylic Acid Acts Upstream of Auxin and Nitric Oxide (NO) in Cell Wall Phosphorus Remobilization in Phosphorus Deficient Rice

**DOI:** 10.1186/s12284-022-00588-y

**Published:** 2022-08-03

**Authors:** Qi Wu, Huai-Kang Jing, Zhi-Hang Feng, Jing Huang, Ren-Fang Shen, Xiao-Fang Zhu

**Affiliations:** 1 State Key Laboratory of Soil and Sustainable Agriculture, Institute of Soil Science, Chinese Academy of Science, Nanjing, 210008 China; 2 University of Chinese Academy of Sciences, Beijing, 100049 China; 3 Department of Applied Biological Chemistry, Graduate School of Agricultural and Life Sciences, The University of Tokyo, Tokyo, 1138657 Japan

**Keywords:** Auxin, Cell wall, NO, P deficiency, SA, Translocation

## Abstract

Salicylic acid (SA) is thought to be involved in phosphorus (P) stress response in plants, but the underlying molecular mechanisms are poorly understood. Here, we showed that P deficiency significantly increased the endogenous SA content by inducing the SA synthesis pathway, especially for up-regulating the expression of *PAL*3. Furthermore, rice SA synthetic mutants *pal*3 exhibited the decreased root and shoot soluble P content, indicating that SA is involved in P homeostasis in plants. Subsequently, application of exogenous SA could increase the root and shoot soluble P content through regulating the root and shoot cell wall P reutilization. In addition, − P + SA treatment highly upregulated the expression of P transporters such as *OsPT*2 and *OsPT*6, together with the increased xylem P content, suggesting that SA also participates in the translocation of the P from the root to the shoot. Moreover, both signal molecular nitric oxide (NO) and auxin (IAA) production were enhanced when SA is applied while the addition of respective inhibitor c-PTIO (NO scavenger) and NPA (IAA transport inhibitor) significantly decreased the root and shoot cell wall P remobilization in response to P starvation. Taken together, here SA-IAA-NO-cell wall P reutilization pathway has been discovered in P-starved rice.

**Supplementary Information:**

The online version contains supplementary material available at 10.1186/s12284-022-00588-y.

## Introduction

Phosphorous (P) is an essential macronutrient that affects the plant growth and development (Shukla et al. 2017). It is previously described that P is an important component of tissue molecules such as phospholipids, nucleic acids and adenosine triphosphate (ATP) (Lei et al. 2015). Even a large amount of P exists in soil, the availability of soluble P is still limited due to the sophisticated complexes with other cations (Ali et al. 2019), thus 30% of the world’s arable soils are under P-deficient condition (Carstensen et al. 2018). Therefore, mineral P fertilizers such as rock phosphate, calcium orthophosphates, ammonium phosphates and nitric phosphates are used to increase the crop yields. However, excessive P fertilization contributes to the water eutrophication, soil deterioration and greenhouse gases, which have direct negative impacts on the natural ecosystems and human health (Garske et al. 2021). Thus, breeding new crop varieties with high efficiency in the P utilization is essential for agriculture sustainability in the future.

To adapt to the low availability of P in the soils, two main strategies have been employed for the survival in plants. First, plants can increase the absorptive capacity of external P through remodeling the root system architecture (Xu et al. 2020), producing the rhizosphere exudates (Canarini et al. 2019) and symbiosis with the mycorrhizal (Kobae et al. 2019). For example, P deficiency reduces primary root length and increases the number of lateral roots (Kawa et al. 2016), secretes the exudates such as hydrogen ion, organic acid, acid phosphatase and secondary metabolites to modify the physical and chemical properties of the soils (Hinsinger et al. 2003; Pant et al. 2015; Wu et al. 2018; Ma et al. 2021), forms symbiotic associations with rhizosphere microorganisms (Campos et al. 2018), all of which facilitate the release of the insoluble P from the soil and make it available for the plants. Another strategy is to increase the reutilization of the P in the plants. For instance, vacuole acts as the major intracellular reservoir for excess P and is important to maintain the P homeostasis in response to changes of P availability in soil. Recent study has identified vacuolar P transporter PHT5 (Phosphate Transporter 5 family) with both the SYG1/PHO81/XPR1 (SPX) domain and major facilitator superfamily (MFS) domain, acts as a P influx to sequester excess P into the vacuolar in *Arabidopsis* (Liu et al. 2016a, b). Conversely, a pair of vacuolar P efflux transporters OsVPE1 and OsVPE2 (Vacuolar GlpT Proteins) in rice are also identified to release P from the vacuole to the cytoplasm in plants to adapt to low P stress (Xu et al. 2019). In addition to vacuole, cell wall, especially pectin, is recognized as a P pool to facilitate the remobilization of the P deposited in cell wall under P starvation (Zhu et al. 2015). Subsequently, a nitric oxide (NO)-ethylene-pectin P remobilization regulatory pathway has been identified in P deficient rice (Zhu et al. 2017).

Salicylic acid (SA), an important phytohormone, is synthesized from the chorismate through isochorismate synthase (ICS) and phenylalanine ammonia-lyase (PAL) pathway (Lefevere et al. 2020). SA is well-known to be involved in the adaptive process of abiotic stress (Pál et al. 2013; Khan et al. 2015), such as salinity stress, ozone stress (Naeem et al. 2020), UV-B radiation (Escobar et al. 2019), temperature stress (Otálora et al. 2020) and drought stress (Khalvandi et al. 2021). In rice, PAL pathway seems to be more important for the accumulation of SA than the ICS pathway. In most cases, *PAL* genes exhibit constitutive expression in plants and differentially express in different tissues when in response to stress response. For example, co-localization with disease resistance QTLs of *PAL *1*–*7 indicates their role in plant defense (Tonnessen et al. 2015). Mutation of *PAL*6 leading to a 70–77% decrease in PAL activity, displaying an increased susceptibility to *M. oryzae* (Duan et al. 2014). *PAL*4, a homologous to *PAL*6, is also shown to be involved in the protection from pathogens invasion (Tonnessen et al. 2015). Abnormal Inflorescence Meristem1 (AIM1), a key enzyme in the PAL pathway, loss of function in *aim*1 mutant results in the reduced SA level and root meristem activity in rice, and this phenotype could be rescued by SA exogenous application (Xu et al. 2017). Further analysis shows a twofold decrease in benzoic acid (BA) content and sixfold increase in t-coumaric acid (tCA) in rice *aim*1 mutants, suggesting that AIM1 may catalyze the conversion of tCA to BA in rice. However, further function of *AIM*1 in response to pathogen infection is still unknown which needs further investigation (Lefevere et al. 2020). In addition, SA is also involved in the adaption to the nutrient deficiency/stress in plants (Wang et al. 2011). For example, SA is shown to respond to Fe-deficiency through the bHLH38/39-controlled transcriptional regulation of the downstream genes in *Arabidopsis* (Shen et al. 2016). In rice, SA alleviates the arsenic (As) toxicity by reducing its translocation from root to shoot (Singh et al. 2015). In soybean, exogenous SA alleviates the side effects of Aluminum (Al) toxicity through modulating the cellular H_2_O_2_ level and the antioxidant enzyme activities (Liu et al. 2017). Although established mechanisms of the involvement of SA in the regulation of nutrients deficiency or stress have been proposed, the interaction between SA and P deficiency is still little understood.

In addition, SA is also demonstrated to respond to the environmental stress via the interaction with some other signal molecules such as nitric oxide (NO) and ethylene (Mur et al. 2013). For example, SA alleviates the inhibition of root elongation under Al toxicity by decreasing the production of ethylene in rice (Zhu et al. 2020). Similarly, interactive effects of SA and NO precursor (SNP) is observed in Cd stressed rice (Mostofa et al. 2019). Nevertheless, it is poorly elucidated the connection between SA and other phytohormones or signal molecules in maintain the P homeostasis in P deficient condition.

In this study, the effect of P deficiency on root and shoot SA content in rice were first investigated. Further analysis demonstrated that SA was involved in the cell wall P reutilization in P deleted condition, and this regulation was dependent on the IAA-NO signal cascade. In conclusion, our study proposes the novel insight of SA-IAA-NO regulatory pathway in regulating the cell wall P remobilization in P deficient rice.

## Materials and Methods

### Plant Materials and Growth Conditions

Rice (*Oryza sativa*) sp. *japonica* ‘Nipponbare’ (Nip) and *indica* ‘Kasalath’ (Kas) cultivars were used in the initial SA content measurement experiment, then Nip was used for the following experiments. Seeds were first sterilized by 10% Bleach solution for 30 min and washed with deionized water for 3 times. After that, seeds were geminated in the incubator at 30 ºC for 2 d. Seeds with similar buds were selected to cultivate in the complete nutrient solution (+ P) for 2 weeks as described in Zhu (Zhu et al. 2015). Then the seedlings were transferred to P-sufficient solution (+ P) or P-insufficient solution (− P) with or without SA supply for 7 d. The pH of the nutrient solutions was adjusted to 5.5. Nutrient solutions were updated per 3 d. All of experiments were conducted in a growth chamber with a 16 h/30 °C day and an 8 h/30 °C night regime, a light intensity of 400 μmol m^–2^ s^–1^ and a relative 60% humidity. All experiments in this study were performed independently at least 3 times.

To explore interactions among SA, auxin and NO under P starvation, two-week buds were transplanted to + P, + P + SA (0.01 mM), − P, − P + SA (0.01 mM) in the presence or absence of 10 μM c-PTIO or 1-N-Napthylphthalamic acid (5 μM NPA). Or two-week buds were transplanted to + P, − P, + P + SNP (2.5 μM), − P + SNP (2.5 μM), + P + NAA (0.05 μM), − P + NAA (0.05 μM) for the measurement of SA content.

*Arabidopsis thaliana* (Columbia ecotype, Col-0, WT) and SA biosynthesis defective mutants *sid*2*-*1* and sid*2*-*2 were obtained from Arabidopsis Biological Resource Centre (ABRC). For the Petri dish experiment, seeds were first sterilized by 5% Bleach solution for 10 min and washed with deionized water for 3 times. The seeds were directly germinated in the agar solidified medium containing P-sufficient solution (+ P) or P-insufficient solution (− P) according to Murashige-Skoog salts (Murashige and Skoog. 1962). The Petri dishes were placed at 4 °C refrigerator for the vernalization for 2–3 d. The growth chamber for *Arabidopsis* was set as a temperature of 23 °C, a light intensity of 140 μmol m^–2^ S^–1^ and a 16/8 h day/night rhythm.

### Plasmid Construction and Plant Transformation

The target site was designed for knock out of *PAL*3 gene using the CRISPR/Cas9 system. Briefly, the spacer to customize sgRNA was cloned by annealing the oligos and then ligated into BbsI-linearized entry vector. Then the entry vector was assembled to the pUBQ-zCas9i destination vector through gateway reaction. The pUBQ-zCas9i destination vector loading sgRNA was used for genetic transformation via the Agrobacterium-mediated transformation (strain EHA105) method for generating transgenic rice, according to Japonica rice transformation methods (Hiei et al. 1997). The primers used in this study are listed in Additional file 1: Table S1.

### SA Content Measurement

The extract protocol was modified according to previous description (Zhang et al. 2018). Around 0.5 g root samples were harvested and ground in the pre-cooled mortar containing 10 mL extraction buffer (isopropanol:water:hydrochloric acid, 2:1:0.002 v/v). The mixture was shaken at 4 °C for 30 min and 20 mL dichloromethane was added again for another shaking at the same condition. Then the supernatant was discarded after centrifuging at 13,000 × g for 5 min. After the extraction of the organic phase, the pellets were dissolved with 150 μL methanol (0.1% methane acid) and filtered with a 0.22 μm filter membrane. Finally, the purified samples were analyzed by high-performance liquid chromatography-tandem mass spectrometry (HPLC–MS/MS). The injection volume was 2 μL. Mass spectometry conditions were as follows: the spray voltage was 4500 V; 15, 65, and 70 psi air curtain, nebulizer, and aux gas were set respectively, and 400 °C atomizing temperature.

### IAA Content Measurement

About 50 mg fresh roots and shoots were ground into power with liquid nitrogen, and then dissolved in 2 mL 0.01 M phosphate buffer (KH_2_PO_4_–NaOH with 0.02% (w/v) ascorbic acid). After the centrifugation, 50 μL supernatant was mixed with 50 μL chromogenic agent in ELISA kit and incubated at 37 °C for 10 min, finally the absorbance at 450 nm was detected.

The procedure of IAA measurement was performed in accordance with instruction in ELISA kit.

### PAL Activity Assay

For the extraction of sample. Around 1–2 g sample was harvested and ground in the pre-cooled mortar containing 10 mL extraction buffer (0.05 mol·L^−1^ boric acid buffer; 5.0 mmol·L^−1^ mercaptoethanol, 1.0 mmol·L^−1^ EDTA-2Na, 5% glycerol pH8.3; 5% polyvinylpyrrolidone). Finally, 4 layers of gauze was used to filtrate the mixture solution and the supernatant was collected by centrifuging at 13,000 × g for 15 min at 4 °C, the supernatant was referred as the tested solution and stored at 4 °C.

For the determination of PAL enzyme activity. 1 mL above mentioned tested solution was mixed with 1 mL 0.02 mol·L^−1^ L-phenylalanine and 2 mL boric acid buffer (pH 8.8). After shaking well, incubated it at 30 °C in water bath for 60 min, 0.2 mL 6 mol·L^−1^ HCl was subsequently added to stop the reaction, then the absorbance at 290 nm was detected.

### Quantitative Real‑Time PCR (qRT‑PCR)

Fresh roots and shoots were harvested in liquid nitrogen and used to extract total RNA immediately by using the TRIzol reagent. The quality and integrity of the RNA was detected through agarose gel electrophoresis. Then 500 ng RNA was used to reverse transcription to cDNA through PrimeScript RT reagent kit. The quantitative real‑time PCR was performed as described before (Zhu et al. 2017). The PCR reaction mixture contains 1 μL cDNA, 1 μL forward primer and reverse primer respectively, 5 μL SYBR Premix (Toyobo, Japan) and 2 μL RNA-free water. Relative expression of the genes was calculated by the 2^−ΔΔCT^ method (Livak et al. 2001) and *OsACTIN* was used as a reference gene. Four biological replicates were used for each treatment, and the primers used in this study are listed in Additional file 1: Table S1.

#### Extraction of Cell Wall Enriched Fractions

To extract cell wall materials, root and shoot samples were ground with liquid nitrogen, 75% ethanol was added to incubate for 20 min in ice, followed by the centrifugation at 13,000 × g for 10 min and removed the supernatant. The pellets were then homogenized with acetone, chloroform:methyl alcohol (1:1) and methyl alcohol respectively, each was washed for 20 min, and centrifuged at 13,000 × g for 10 min to discard the supernatant. The remaining residue, regarded to as cell wall materials, was stored in a 4 °C freezer for the following experiment.

To extract cell wall pectin, around 2 mg cell wall materials were weighed and mixed with 1 mL deionized water for the incubation in water bath at 100 °C for 1 h. Then the supernatant was collected after the centrifugation and transferred to a new 5 mL tube. This step was repeated for another two times and combined supernatant solution was referred as pectin for further analysis.

### Determination of P Content

To determine the P retention in cell wall, around 5 mg cell wall materials were weighed and then 1 mL 2 M HCl was added to incubate for 3 d with shaking continuously. The samples were centrifuged at 13,000 × g for 20 min to collect the supernatant, and the P content in the supernatant was determined by inductively coupled plasma atomic emission spectroscopy (ICP-AES; Fisons ARL Accuris, Ecublens, Switzerland).

To measure the P deposition in cell wall pectin, 1 mL above extracted pectin was diluted to total 10 mL volume by adding additional deionized water, P content in diluted pectin solution was determined by ICP-AES.

To determine the root/shoot soluble P content in rice and *Arabidopsis*, fresh roots and shoots were harvested, washed and weighed. The samples were then homogenized in liquid nitrogen and incubated with 4 mL of 5% (v/v) 5 M H_2_SO_4_ solution for 2 h with shaking. After centrifuging at 13,000 × g for 10 min, 400 µL supernatant was transferred to a 96-well microplate and mixed with 200 μL ammonium molybdate containing 15% ascorbic acid (w/v; pH 5.0) for the incubation in 37 °C for 30 min. Finally, the absorbance at 650 nm was recorded and P content was normalized using fresh weight.

To measure the root and shoot total P content in rice, rice seedlings were washed three times with deionized water and roots and shoots were harvested separately, then the samples were weighed and mixed with 2 mL HNO_3_ for the digestion at 120 °C for 24 h. Digested samples were diluted with 18.2 MΩ water and analyzed by ICP-AES.

For collection of xylem sap, rice shoots were first excised with a razor (2 cm above the roots), then collected the rising xylem sap for 2 h after the shoot decapitation, the collected xylem juice was finally diluted with 1 mL deionized water (Che et al. 2016). P content in xylem sap was measured by ICP-AES.

### Uronic Acid Measurement

Pectin content in cell wall was referred as the uronic acid according to Blumenkrantz (1973). In brief, 1 mL 98% H_2_SO_4_ (containing 12.5 mM Na_2_B_4_O_7_⋅10H_2_O) was added to the 200 µL above extracted pectin solution and incubated at 100 °C for 5 min, then 20 μL 0.15% M-hydroxy-diphenyl was mixed and let stand 30 min at the room temperature, the absorbance at 520 nm was measured and galacturonic acid was used as the standard.

### Determination of Pectin Methylesterase (PME) Activity

The PME activity was detected as the descried protocol previously with the minor modifications (Zhu et al. 2017). Approximate 3 mg above extracted cell wall was weighed and homogenized in 1 M NaCl solution (dissolved in 10 mM Tris buffer, pH 6.0) at 4 °C with shaking for 1 h. The mixture solution was centrifuged at 13,000 × g for 20 min to collect the supernatant. Then 50 µL collected supernatant was transferred to the microplate together with 100 µL 200 mM PBS solution (containing 640 µg mL^−1^ pectin pH 7.5) and 10μL alcohol oxidase for the incubation at room temperature for 10 min, finally 200 µL 0.5 M NaOH (including 5 mg mL^−1^ Purpald) was added and absorbance was measured at 550 nm (methanol was used as the standard).

### Endogenous NO Detection in Roots

NO signal in roots was detected using 10 µM 4-amino-5-methylamino-2,7-difluorofluorescein diacetate (DAF-FM DA). 1 cm root tip region was excised with a razor and washed with HEPES–KOH (pH 7.4) for 15 min. Then the samples were incubated in 500 µL DAF-FM DA dye in the dark for 30 min, rinsed the root tips with fresh HEPES–KOH buffer for three times to eliminate auto-fluorescence. NO signal fluorescence was captured using Nikon Eclipse 80i light microscope. The fluorescence intensity was quantified by Image J software (https://imagej.nih.gov/ij).

### Statistical Analysis

All experiments were performed at least three times. One-way ANOVA was used to analyze the data, and the mean values were compared using Duncan’s multiple range test. Different letters above figures indicate that the mean values were statistically different at *P* < 0.05 level.

## Results

### P Deficiency Enhances SA Accumulation

**Fig. 1:**
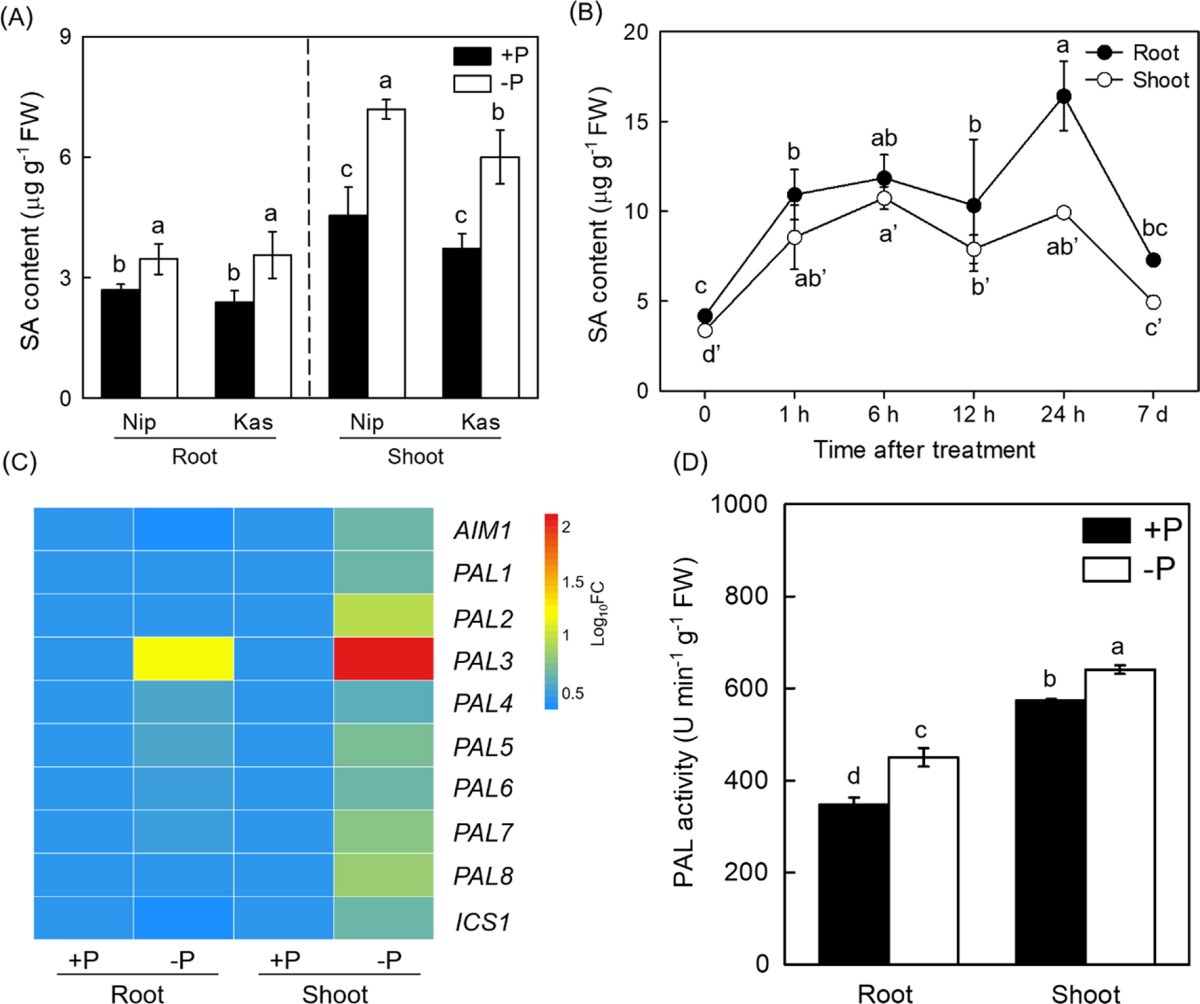
P deficiency activates SA accumulation in rice. Root and shoot SA content (**A** and **B**), SA biosynthetic genes expression (**C**) and PAL activity (**D**) under –P treatment for 7 d in rice were measured. Data are means ± SD (*n* = 4). Different letters represent significant differences by Duncan’s multiple range test at the *P* < 0.05

To investigate the effect of P deficiency on SA accumulation, root and shoot SA content were measured under P-sufficient or P-insufficient condition in both Nip and Kas cultivars. As shown in Fig. 1A, SA content was significantly increased in roots and shoots in both cultivars, especially in Nip, after 24 h P-deficient treatment (Fig. 1B). Then to understand how the SA accumulation was affected, the expression of the critical genes such as *OsICS* (*Isochorismate Synthase*), *OsPAL* (*Phenylalanine Ammonia-lyase*) and *OsAIM*1 (*Abnormal Inflorescence Meristem*1), that are responsible for SA biosynthesis in rice were investigated (Lefevere et al. 2020). As shown in Fig. 1C P deficiency significantly induced the expression of several *PAL* genes, especially for the *PAL*3, which was further confirmed by the elevated PAL activity in both root and shoot (Fig. 1D), implying that P deprivation could enhance the SA accumulation through the SA biosynthesis related PAL pathway in rice.

### Exogenous SA Alleviates P Deficiency

**Fig. 2:**
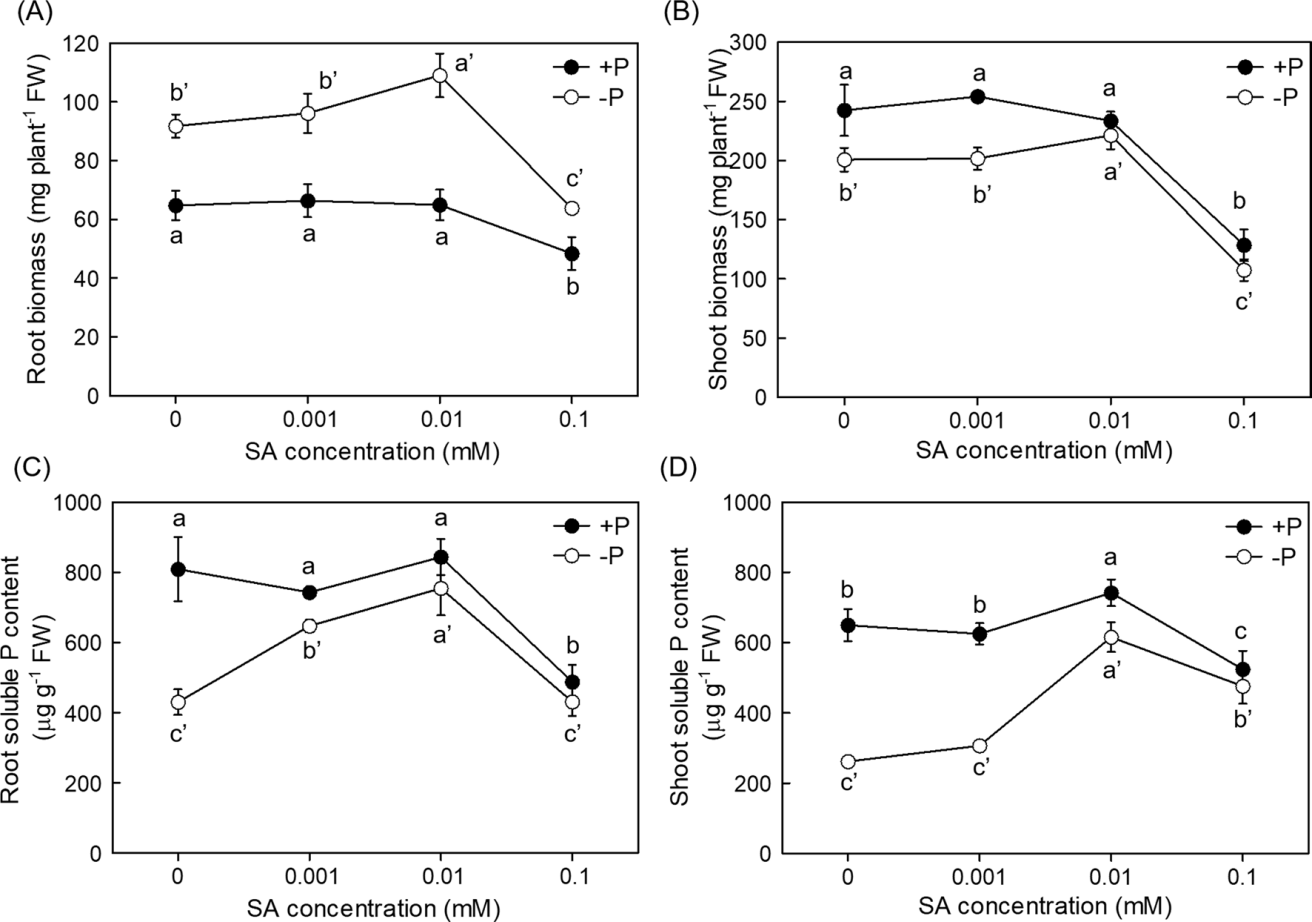
The effect of different concentrations of SA on the plant growth under P deficient condition in rice. Root and shoot biomass (**A** and **B**) and soluble P content (**C** and **D**) in Nip were measured. Data are means ± SD (*n* = 4). Different letters represent significant differences by Duncan’s multiple range test at the *P* < 0.05

P deficiency inhibited root growth in rice (Negi et al. 2016), to explore the influence of SA on the P deficient rice, root and shoot biomass were measured after SA treatment with different concentrations. As shown in Fig. 2A, B, P starvation resulted in the increased root but decreased shoot biomass, while the biomass was alleviated in the presence of 0.01 mM SA application under P deficiency, suggesting the positive role of the SA when rice in response to P deficiency. Moreover, the decreased biomass defect was aggravated when 0.1 mM SA was applied, indicating excess SA content may also cause a side effect on plant growth.

Then, a question raised whether the improved growth under − P + SA treatment was resulted from the sufficient P supply in plants. To test this possibility, root and shoot soluble P content under different SA concentrations were measured. As shown in Fig. 2C, D, the application of 0.01 mM SA could significantly increase the root and shoot soluble P content under P deficiency, indicating that exogenous SA could mitigate the P deficiency through increasing the root and shoot soluble P content.

### Decreased Endogenous SA Synthesis Aggravates P Deficiency

**Fig. 3:**
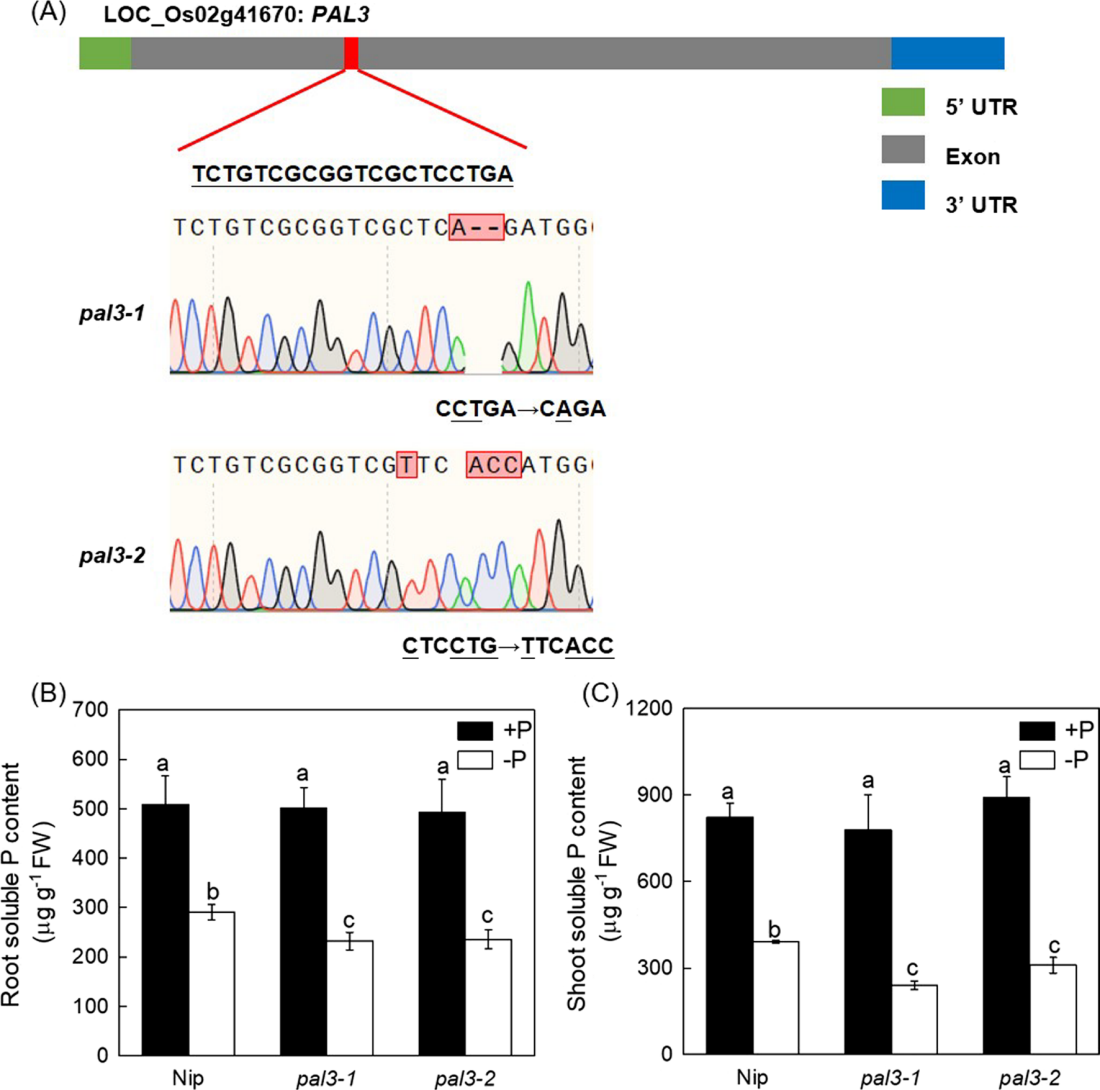
The effect of endogenous SA on the soluble P content in rice. (**A**) *PAL*3 target loci. The gRNA targeting site was designed in the exon of *PAL*3, two kinds of mutations were generated using CRISPR-Cas9 technology. Root and shoot soluble P content (**B** and **C**) in rice were measured. Data are means ± SD (*n* = 4). Different letters represent significant differences by Duncan’s multiple range test at the *P* < 0.05

As the expression of *PAL*3 is significantly induced under P deficiency (Fig. 1C), then to further understand the effect of SA on P deficiency, we generated two independent lines of knock out of *PAL*3 gene using CRISPR/Cas9 system (Fig. 3A). It was interesting that both root and shoot soluble P content were decreased in *pal*3 mutants under P deficiency compared with Nip (Fig. 3B, C), indicating that endogenous SA level may attribute to soluble P content in adaption to P deficiency. In addition, AtICS is require for the synthesis of the SA in *Arabidopsis* (Lefevere et al. 2020). two mutants *sid*2*-*1 and *sid*2*-*2 that are deficient in SA synthesis in *Arabidopsis* were also used. Endogenous SA content was decreased in the *sid*2 mutants (Additional file 1: Fig. S1), compared with WT, the *sid*2 mutants exhibited more inhibited root growth in P deficient condition (Additional file 1: Fig. S2A, B), in company with the decreased root and shoot soluble P content (Additional file 1: Fig. S2C, D), suggesting that decreased endogenous SA synthesis attributed to the more sensitive P deficiency phenotype in the *sid*2 mutants.

### Exogenous SA Enhances the Reutilization of the Cell Wall Retained P

**Fig. 4:**
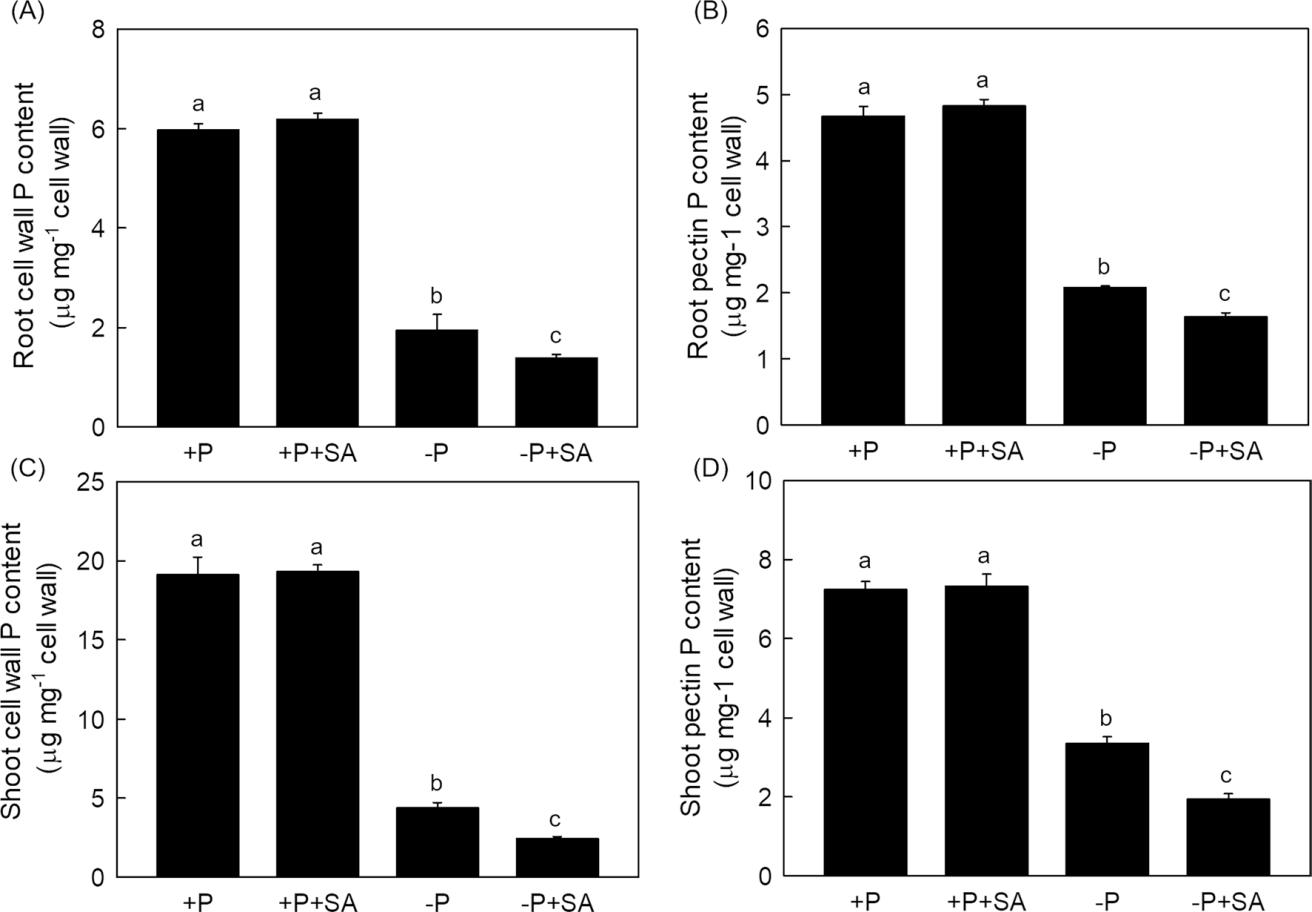
SA affects the root and shoot cell wall reutilization in rice. Cell wall P content in root (**A**) and shoot (**C**), pectin P content in root (**B**) and shoot (**D**) were measured. Data are means ± SD (*n* = 4). Different letters represent significant differences by Duncan’s multiple range test at the *P* < 0.05

Cell wall accounts 50% of the total P in the rice root, and pectin has been demonstrated to have the ability to reutilize the P retained in the cell wall (Zhu et al. 2015). Thus, a question raised whether root cell wall P reutilization is also involved in the process of SA-alleviated P deficiency. To test this hypothesis, root cell wall was extracted and the P deposited in cell wall was measured. As displayed in Fig. 4A, B, compared with P deficiency alone, P content in both root cell wall and pectin were decreased under − P + SA treatment, indicating that SA promotes the release of the P from the cell wall. It is noteworthy that root pectin content was not changed whereas PME activity was significantly increased after SA was applied (Additional file 1: Fig. S3A, B), suggesting that SA could facilitate the reutilization of the cell wall deposited P, rather than altering the pectin itself. Unexpectedly, cell wall and pectin P content in shoot were also reduced under –P + SA treatment (Fig. 4C, D) whereas no changes were observed in shoot pectin content and PME activity (Additional file 1: Fig. S3C, D), which still needs to be investigated. In summary, our results mainly demonstrated that SA enhances the cell wall P reutilization in P-deficient rice.

### SA Increases the Expression of Genes Responsible for P Translocation

**Fig. 5:**
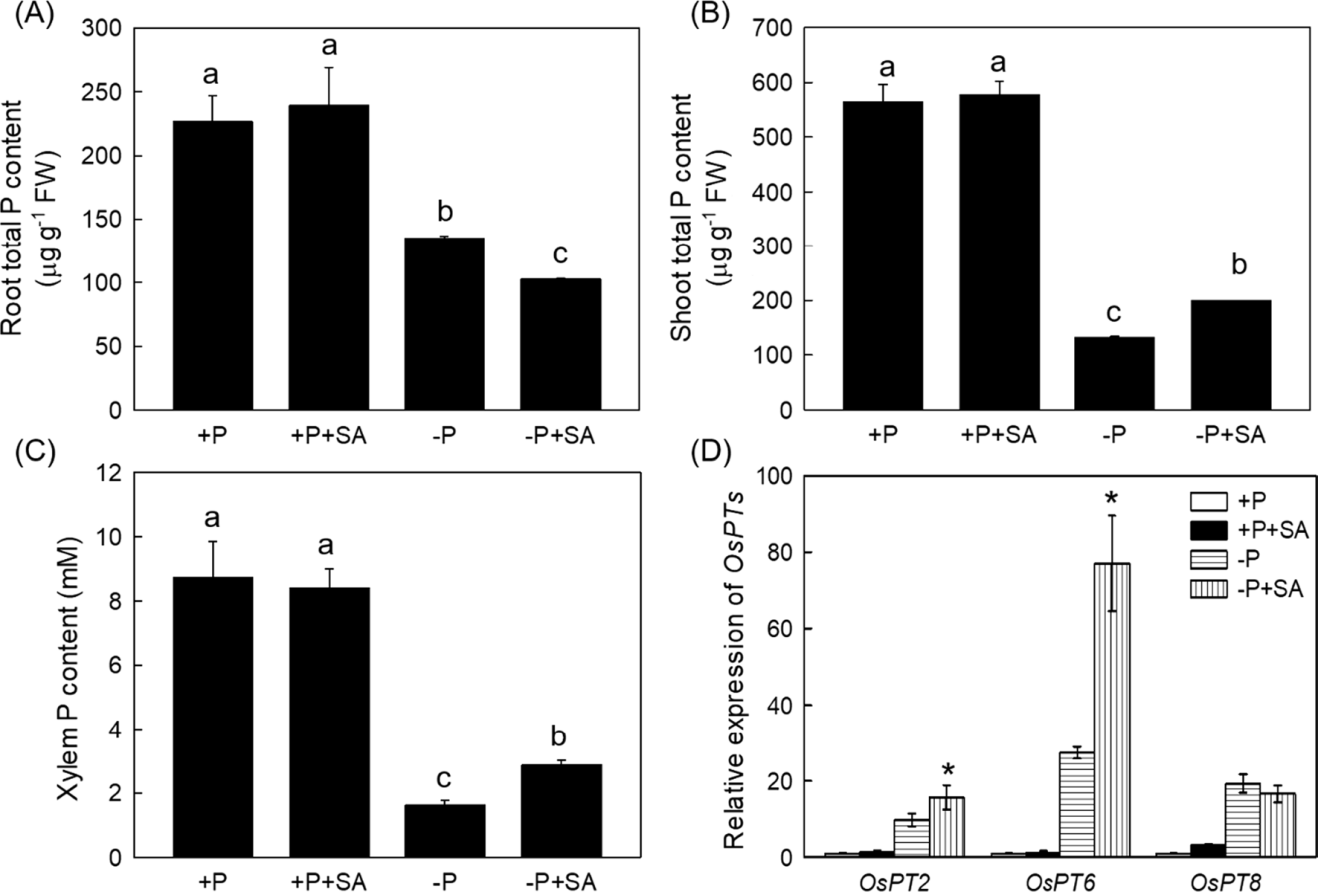
SA facilitates the P translocation under P-insufficient condition in rice. Root and shoot total P content (**A** and **B**), xylem P content (**C**) and the relative expression of P related transporter genes were detected. Data are means ± SD (*n* = 4). Different letters represent significant differences by Duncan’s multiple range test at the *P* < 0.05 and asterisks indicate a significant difference at *P* < 0.05 by Student’s t test

Previous studies demonstrated that increased root soluble P content originated from the cell wall deposited P contributed to increased translocation of the P from root to the shoot (Zhu et al. 2018). To confirm this assumption, root and shoot total P content were measured. As expected, compared with P deficiency alone, − P + SA treatment significantly decreased the total P content in root and significantly increased the total P content in shoot (Fig. 5A, B), in company with the increased P content in xylem (Fig. 5C), indicating that SA could facilitate the P translocation from root to shoot. Thus the expression of genes involved in P translocation was investigated. As shown in Fig. 5D, the expression of *OsPT*2 and *OsPT*6, both encoding key transports for root-to-shoot P translocation (Ai et al. 2009), were higher under − P + SA treatment than that in − P condition alone, suggesting that *OsPT*2 and *OsPT*6 were responsible for SA stimulated root to shoot P translocation under P-deficient condition. In addition, several *SPX* (*SYG*1*/PHO*81*/XPR*1) genes which are responsive to P starvation (Wang et al. 2014a, b, c; Shi et al. 2014), are highly induced by − P treatment, and were partially reversed by the addition of SA (Additional file 1: Fig. S4), further confirming the role of SA in alleviating the P deficiency limited plant growth.

### SA Stimulates Both NO and Auxin Accumulation Under P Deficiency

**Fig. 6:**
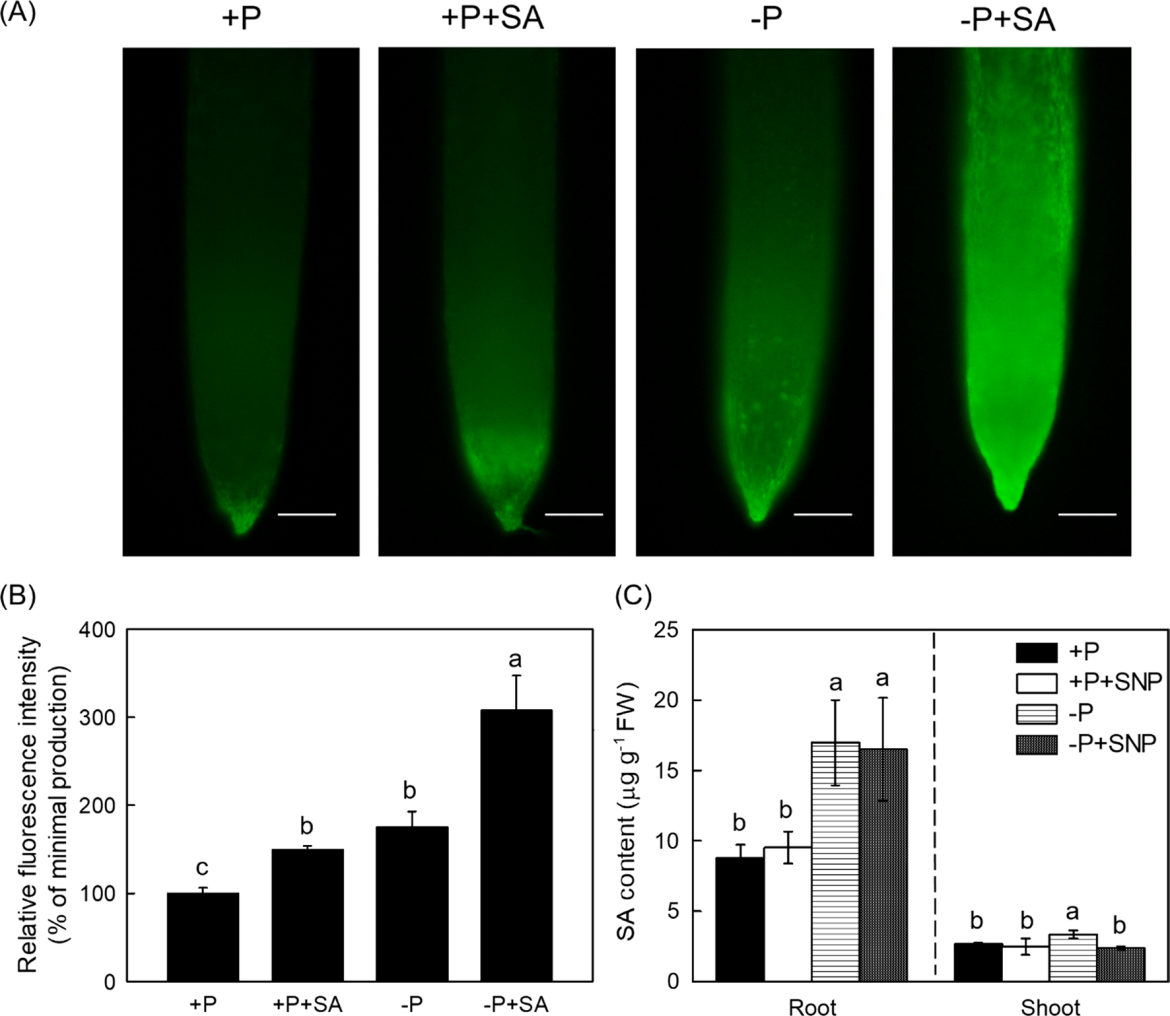
SA enhances the NO production in response to P deficiency in rice. Root endogenous NO staining (**A**) indicated by green fluorescence and NO production (**B**) described as relative fluorescence intensity (% of minimal production) under respective conditions were displayed. Root and shoot SA content (C) in P-limited condition with or without SNP supply were measured. Data are means ± SD (*n* = 4 for SA content measurement, *n* = 8 for root NO detection). Different letters represent significant differences by Duncan’s multiple range test at the *P* < 0.05. Scale bar = 1 mm

**Fig. 7:**
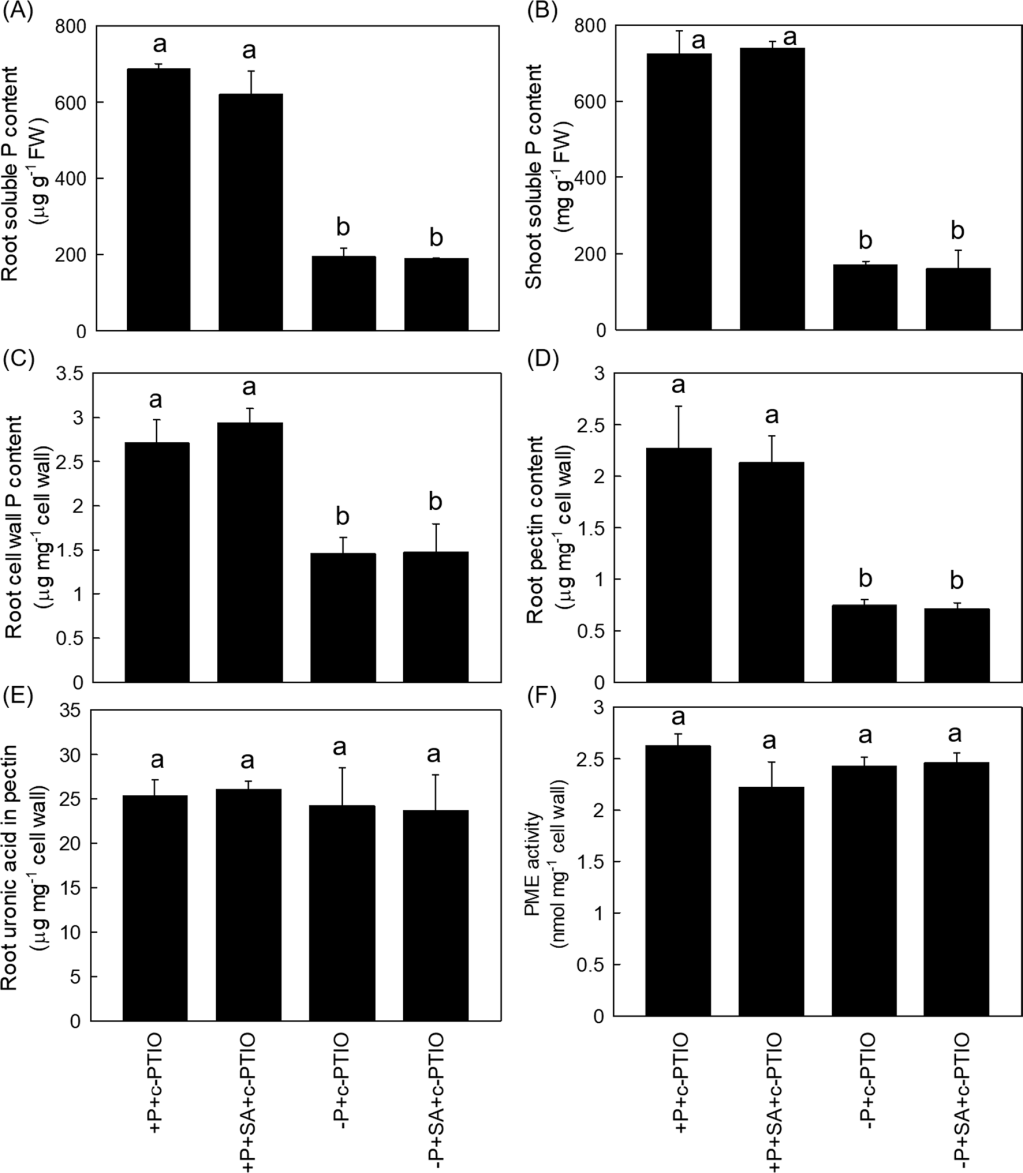
SA promoted root cell wall P reutilization in rice is dependent on NO. Soluble P content in root (**A**) and shoot (**B**), root cell wall P content (**C**), cell wall pectin P content (**D**), root pectin content (**E**) and PME activity (**F**) were measured. Data are means ± SD (*n* = 4). Different letters represent significant differences by Duncan’s multiple range test at the *P* < 0.05

NO acts upstream of ethylene in cell wall P reutilization process in P-deficient rice (Zhu et al. 2017), thus the relationship between NO and SA was detected, as shown in Fig. 6, exogenous SA induced the accumulation of NO in P-insufficient root (Fig. 6A, B), whereas the addition of NO donor (SNP) could not affect the endogenous SA content significantly (Fig. 6C). In addition, compared to Nip cultivar, it was interesting that root NO content was decreased in *pal*3 mutants which endogenous SA level is much lower (Additional file 1: Fig. S5). Taken together, these results indicate that NO might act downstream of SA in response to P deficiency. To further confirm this conclusion, the root and shoot soluble P content, cell wall and pectin P content, pectin content and PME activity were all measured after the application of c-PTIO (NO inhibitor). As expected, with the addition of the c-PTIO, the SA-mediated root and shoot cell wall P remobilization was abolished (Fig. 7 and Additional file 1: Fig. S6), indicating that the effect of SA on the cell wall P reutilization is dependent on NO.

**Fig. 8:**
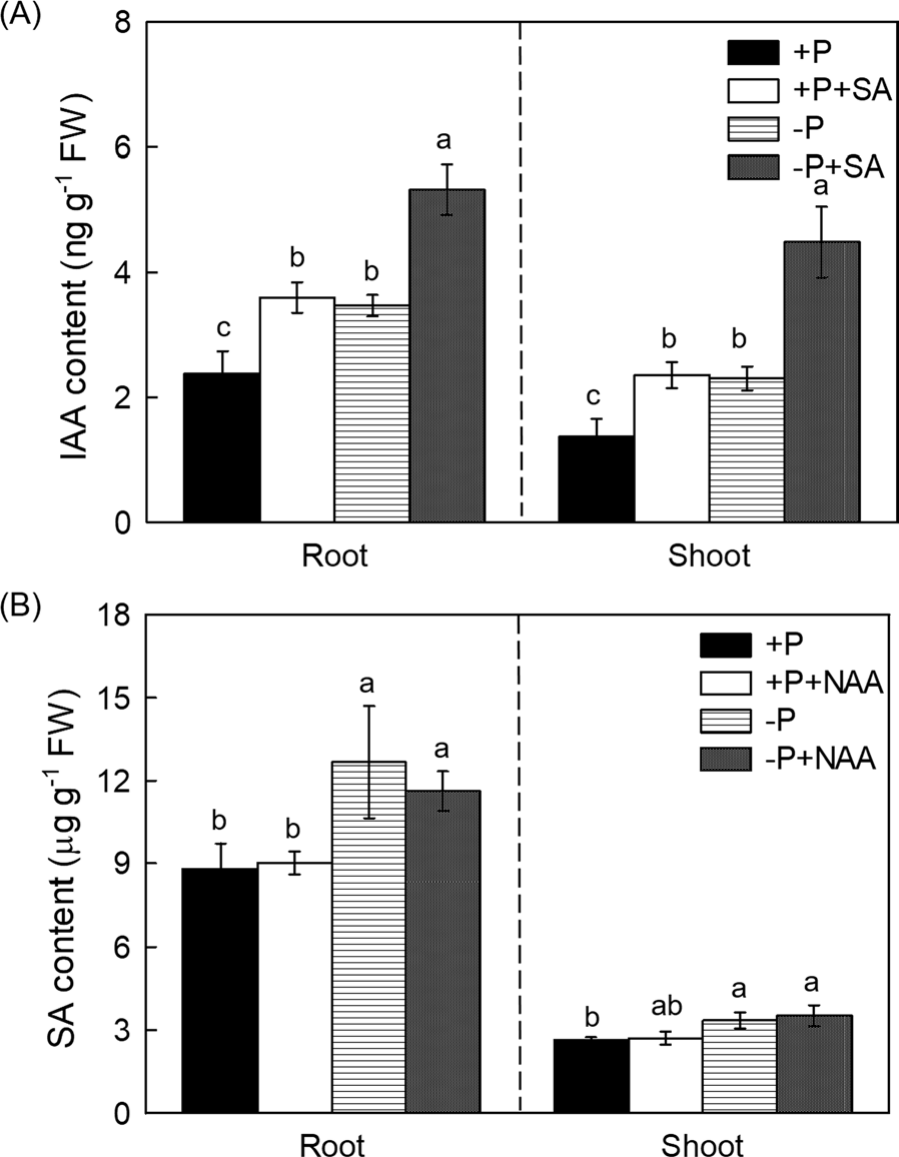
SA increases the root and shoot IAA content in response to P starvation in rice. IAA (**A**) and SA content (**B**) in root and shoot under different conditions were measured. Data are means ± SD (*n* = 4) and different letters represent significant differences by Duncan’s multiple range test at the *P* < 0.05

**Fig. 9:**
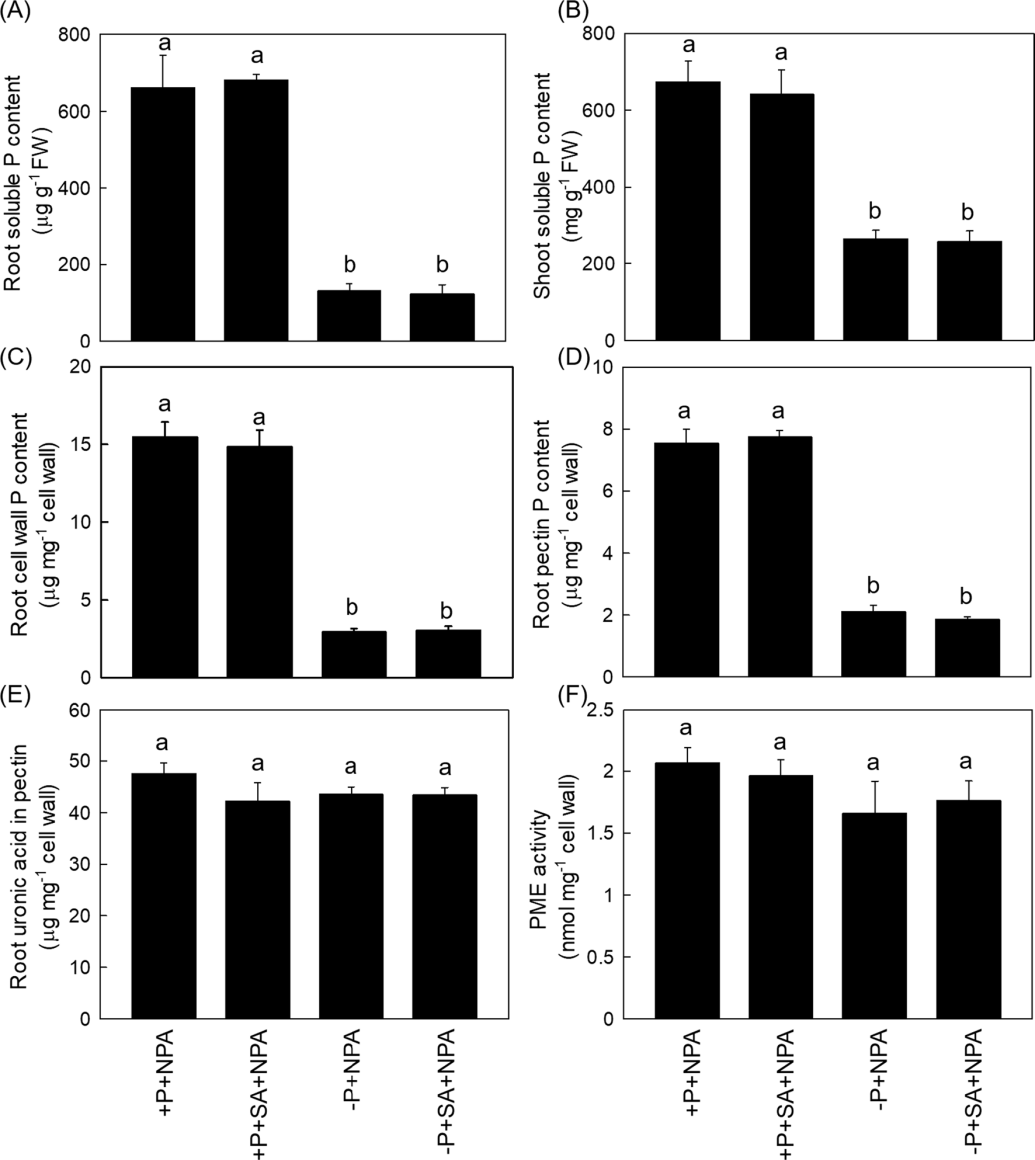
SA mediated root cell wall P reutilization in rice is dependent on auxin. Soluble P content in root (**A**) and shoot (**B**), root cell wall P content (**C**), cell wall pectin P content (**D**), root pectin content (**E**) and PME activity (**F**) were measured. Data are means ± SD (*n* = 4). Different letters represent significant differences by Duncan’s multiple range test at the *P* < 0.05

Our recent study has also displayed that auxin (IAA) can act upstream of NO in affecting the root cell wall P reutilization in P deficient rice (Huang et al. 2022), to understand whether SA acts dependently in auxin-NO mediated pathway or not, endogenous auxin content was detected. It is interesting that the auxin content was increased after SA was applied exogenously (Fig. 8A), while the SA content was not affected after the addition of the NAA (auxin donor, an analogy of IAA) (Fig. 8B). Moreover, in comparison with Nip cultivar, both root and shoot auxin content were detected to be significantly decreased in endogenous SA-deficient *pal*3 mutans (Additional file 1: Fig. S7), further implying that SA might also act upstream of auxin in regulating the cell wall P remobilization process in the P deficient rice. Finally, this hypothesis was further confirmed by the reversed effect of NPA in the SA induced alteration of root and shoot soluble P content, cell wall and pectin P content (Fig. 9 and Additional file 1: Fig. S8).

## Discussion

SA has previously been demonstrated to participate in the alleviation of abiotic stress such as iron (Fe) deficiency (Shen et al. 2016; Jelali et al. 2021), Al toxicity (Zhu et al. 2020), potassium (K) deficiency (Zhu et al. 2021), cadmium (Cd) stress (Liu et al. 2016a, b) and salt stress (Ma et al. 2017) in plants. Recently, Wang et al. demonstrated that the SA-mediated pathogenic response, was affected by one phosphate transporter gene *PHT*4*;*1*,* which was independent of known SA regulators like ADL1, EDS5 and PAD4 (Wang et al. 2014a, b, c). However, little information is known about the effect of SA on P deficiency and its underlying molecular mechanism still remains elusive. In this study, we demonstrated that P deficiency increased root and shoot SA level, which attributed to the increased PAL activity by increasing the expression of genes that responsible for the SA biosynthesis, especially for *PAL*3 (Fig. 1). On the other hand, we generated the SA synthetic mutants of *PAL*3 gene, which is significantly responsive to P deficiency, in *pal*3 mutants, both root and shoot soluble P content were decreased (Fig. 3), while SA applied exogenously significantly increased the root and shoot soluble P content in rice and mitigated the P deficiency (Fig. 2). Collectively, these results indicate that the accumulation of SA induced by P deficiency takes an important role in the alleviation of P starvation in rice.

Cell wall, which is considered as the first barrier to protect plant cells, plays important roles in response to abiotic stress such as nutrients deficiency, drought, flooding, salinity, and heavy metal contaminants (Houston et al. 2016). Cell wall is composed of pectin, hemicellulose and cellulose (Cosgrove. 2005). In 1996, Ae et al. first demonstrated that root cell walls from groundnut exhibited a higher P-solubilizing activity than other species (Ae et al. 1996). Subsequently, the negatively charged carboxylic groups (–COO–) in pectin was demonstrated to chelate Fe^3+^ or Al^3+^ and then facilitated the insoluble P released from the clay minerals in P-insufficient condition (Gessa et al. 1997). More recently, Zhu et al. first demonstrated that cell wall pectin enhanced P reutilization under P deficient condition in different rice varieties. Specifically, the higher the pectin content, the stronger capability of it to solubilize the P fixed in the cell wall (Zhu et al. 2015). In addition to pectin itself, the modification of pectin such as demethyl-esterification process controlled by PMEs (Pectin Methyl-esterification Enzymes), was also contributed to the cell wall P reutilization process (Wu et al. 2021). Here we displayed that the application of SA decreased the P retention in root cell wall and pectin when external P nutrient was insufficient, in company with the significantly increased PME activity (Fig. 4 and Additional file 1: Fig. S3), indicating that this SA-promoted root cell wall P reutilization pathway is dependent on the PME activity, instead of the pectin content itself. In addition, shoot cell wall P content was also decreased under − P + SA treatment even though almost no variation of pectin content and PME activity were observed in shoot cell wall (Fig. 4 and Additional file 1: Fig. S3), suggesting an unknown cell wall P remobilization pathway may exist in shoots which needs further study. To our knowledge, this is first finding clarified that both root and shoot cell wall P can be reutilized in phosphorus deficient rice which might function in different pathways.

Then, how SA could alleviate P deficiency? Previous studies have proposed that some phytohormones including ethylene, abscisic acid (ABA) and NO are able to facilitate the translocation of the internal soluble P from root to the shoot via the up-regulation of genes that are responsible for long-term transport (Zhu et al. 2017; 2018). Three P transporters (OsPT2, OsPT6 and OsPT8) have been identified in rice (Ai et al. 2009; Jia et al. 2011). For example, OsPT2, a low-affinity P transporter, is highly expressed in vascular bundle cells of primary roots and required for the transport of P (Ai et al. 2009). OsPT6 and OsPT8, broadly express in various tissues including root, shoot, seeds, stamens and caryopses, have a high affinity for P transport and are critical to maintain the P homeostasis in plants (Ai et al. 2009; Jia et al. 2011). Accordingly, SA could markedly up-regulate the expression of *OsPT*2 and *OsPT*6 in P-limited condition (Fig. 5), in accordance with the decreased total P content in root, and increased total P and P concentration in shoot and xylem, respectively (Fig. 5), indicating SA indeed promotes the translocation of the P in P deficient rice. In addition, SPX proteins are reported to contain SPX domains, and 6 homologous genes (named *OsSPX*1*-*6) are identified in rice (Wang et al. 2014a, b, c). Among them, OsSPX1 and OsSPX2, acted as the inhibitors of PHR2 (Phosphate Starvation Response Regulator 2), are critical in the P sensing process (Wang et al. 2014a, b, c). OsPX3 and OsSPX5, function as the repressors of PHR2 to maintain the P homeostasis and signaling (Shi et al. 2014). OsPX6, not only serves as a negative regulator in preventing PHR2 binding to the PSI genes, but also impedes PHR2 translocation into the nucleus in P-replete condition (Zhong et al. 2018). Here we found that P deficiency significantly enhanced the expression of *OsSPX* genes (Additional file 1: Fig. S4), which was consistent with previous study (Zhong et al. 2018). However, − P + SA treatment reduced *OsSPXs* genes expression remarkably compared with − P treatment alone (Additional file 1: Fig. S4), further suggesting the involvement of the SA was in the P starvation signal sensing.

In plants, SA is recognized by its downstream target NPR1 (Nonexpressor of Pathogenesis-Related protein 1), which is phosphorylated and degraded through the proteasome mediated degradation pathway after interacting with other transcription factors (Kumar et al. 2014). Very recently, Wang et al. (2014a, b, c) reported one P transporter PHT4;1, which acted as a SA regulator that working independently of several known SA genes. Nevertheless, it still remains controversial about the signaling networks mediated by SA- in P-limited response. In addition to SA, signal molecular NO has been demonstrated to take part in regulating cell wall P reutilization. For example, NO is reported to act upstream of ethylene to affect the root cell wall P reutilization, a pathway that works independent of ABA (Zhu et al. 2017). Previous studies demonstrate that the increased SA level responds to enhanced NO accumulation (Mur et al. 2013), and other signals such as auxin also plays key roles in the P foraging by the rice roots through promoting hair elongation (Giri et al. 2018). In addition, the crosstalk between auxin and P deficient signaling in rice that is regulated by auxin response factors (AFRs) is also well understood in the past years (Wang et al. 2013). However, the effect of the interaction between SA, NO and auxin on P starvation still remains to be indistinct. In this study, we found − P + SA treatment significantly enhanced the NO accumulation and the auxin content compared with − P treatment alone (Figs. 6 and 8), and this SA-alleviated P deficiency was reversed either through the application of the NO specific inhibitor c-PTIO or by the auxin transport inhibitor NPA (Figs. 7 and 9). All these results indicated that SA improved P nutrition by promoting the root and shoot cell wall remobilization, a process depend on the auxin-NO pathway.

In conclusion, as shown in Additional file 1: Fig. S9, root and shoot SA level is induced by P deficiency, and this − P-induced SA further facilitates the auxin-NO dependent cell wall P reutilization pathway and root to shoot soluble P translocation process, respectively, which may be acted as a great agronomic practice to improve the plant growth in P-limited soils through the addition of the SA.

## Supplementary Information

**Additional file 1: Table S1.** Primers used in this study. **Fig. S1**. The SA content in WT (Col-0) and sid2 mutants. *A. thaliana* WT (Col-0) and sid2 mutants grown in P-sufficient (+P) condition for two weeks and then root SA content was measured. Data are means ± SD (*n*=4) and different letters represent significant differences by Duncan’s multiple range test at the *P*<0.05. **Fig. S2**. The phenotype of WT (Col-0) and the sid2 mutants. *A. thaliana* seedlings grown in P-deficient (− P) or P-sufficient (+P) conditions for 7 d (**A**). Primary root length (**B**), soluble P content in root (**C**) and shoot (**D**) were detected. Data are means ± SD (*n*=4 for soluble P content measurement, *n*>10 for root length observation). Different letters represent significant differences by Duncan’s multiple range test at the *P*<0.05. Scale bar=1 cm. **Fig. S3**. The effect of SA on cell wall pectin content and PME activity in rice. Pectin content (**A** and **C**) and PME activity (**B** and **D**) in root and shoot in the presence or absence of SA under P deficiency were analyzed. Data are means ± SD (*n*=4). Different letters represent significant differences by Duncan’s multiple range test at the *P*<0.05. **Fig. S4**. The effect of SA on P signaling genes in rice under P deficiency. *SPX1, SPX2, SPX3, SPX5* and *SPX6* mRNA level in roots were detected under –P treatment with or without SA supply were quantitated. Data are means ± SD (*n*=4) and asterisks indicate a significant difference at *P*<0.05 by Student’s t test. **Fig. S5**. The effect of endogenous SA on the NO level in rice. Root endogenous NO staining (**A**) indicated by green fluorescence and NO production (**B**) described as relative fluorescence intensity (% of minimal production) in Nip and *pal*3 mutants under respective conditions were displayed (*n*=8). Different letters represent significant differences by Duncan’s multiple range test at the *P*<0.05. Scale bar = 1 mm. **Fig. S6**. SA promoted shoot cell wall P reutilization in rice is dependent on NO. Shoot cell wall P content (**A**), cell wall pectin P content (**B**), shoot pectin content (**C**) and PME activity (**D**) were measured. Data are means ± SD (*n*=4). Different letters represent significant differences by Duncan’s multiple range test at the *P*<0.05. **Fig. S7**. The effect of endogenous SA on the IAA content in rice. Root (**A**) and shoot (**B**) IAA content in Nip and *pal*3 mutants under different conditions were measured. Data are means ± SD (*n*=4) and different letters represent significant differences by Duncan’s multiple range test at the *P*<0.05. **Fig. S8**. SA mediated shoot cell wall P reutilization in rice is dependent on auxin. Shoot cell wall P content (**A**), cell wall pectin P content (**B**), shoot pectin content (**C**) and PME activity (**D**) were measured. Data are means ± SD (*n*=4). Different letters represent significant differences by Duncan’s multiple range test at the *P*<0.05. **Fig. S9**. A working model for SA-alleviated P deficiency in rice deficiency induced-SA accumulation could improve the rice growth via two different pathways. One is to activate root and shoot cell wall P remobilization and then solubilize root and shoot cytoplasm P for the survival, which process is dependent on SA-auxin-NO mediated pathway. The other is to facilitate the root-to-shoot P translocation through upregulating the expression of *OsPT*2 and *OsPT*6.

## Data Availability

All data supporting the findings of this study are available within the paper and within its supplementary materials published online.
