# Supplementary material for: Salicylic Acid Acts Upstream of Auxin and Nitric Oxide (NO) in Cell Wall Phosphorus Remobilization in Phosphorus Deficient Rice
Source: Rice (N Y). 2022 Aug 3;15:42. doi: 10.1186/s12284-022-00588-y (PMC9349334; doi:10.1186/s12284-022-00588-y)
Supplement: Supplementary file 1 — Additional file 1: Table S1. Primers used in this study. Fig. S1. The SA content in WT (Col-0) and sid2 mutants. A. thaliana WT (Col-0) and sid2 mutants grown in P-sufficient (+P) condition for two weeks and then root SA content was measured. Data are means ± SD (n=4) and different letters represent significant differences by Duncan’s multiple range test at the P<0.05. Fig. S2. The phenotype of WT (Col-0) and the sid2 mutants. A. thaliana seedlings grown in P-deficient (− P) or P-sufficient (+P) conditions for 7 d (A). Primary root length (B), soluble P content in root (C) and shoot (D) were detected. Data are means ± SD (n=4 for soluble P content measurement, n>10 for root length observation). Different letters represent significant differences by Duncan’s multiple range test at the P<0.05. Scale bar=1 cm. Fig. S3. The effect of SA on cell wall pectin content and PME activity in rice. Pectin content (A and C) and PME activity (B and D) in root and shoot in the presence or absence of SA under P deficiency were analyzed. Data are means ± SD (n=4). Different letters represent significant differences by Duncan’s multiple range test at the P<0.05. Fig. S4. The effect of SA on P signaling genes in rice under P deficiency. SPX1, SPX2, SPX3, SPX5 and SPX6 mRNA level in roots were detected under –P treatment with or without SA supply were quantitated. Data are means ± SD (n=4) and asterisks indicate a significant difference at P<0.05 by Student’s t test. Fig. S5. The effect of endogenous SA on the NO level in rice. Root endogenous NO staining (A) indicated by green fluorescence and NO production (B) described as relative fluorescence intensity (% of minimal production) in Nip and pal3 mutants under respective conditions were displayed (n=8). Different letters represent significant differences by Duncan’s multiple range test at the P<0.05. Scale bar = 1 mm. Fig. S6. SA promoted shoot cell wall P reutilization in rice is dependent on NO. Shoot cell wall P content (A), cell wa [file 12284_2022_588_MOESM1_ESM.docx]

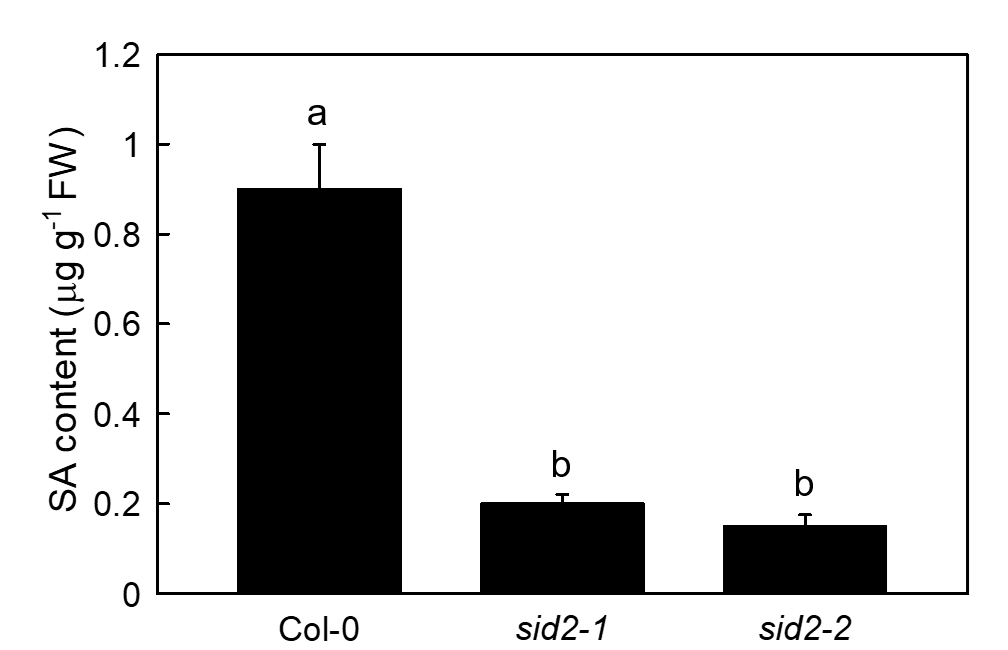


**Supplemental Fig. 1 The SA content in WT (Col-0) and *sid2* mutants**

*A. thaliana* WT (Col-0) and *sid2* mutants grown in P-sufficient (+P) condition for two weeks and then root SA content was measured. Data are means ± SD (n=4) and different letters represent significant differences by Duncan’s multiple range test at the *P*<0.05.


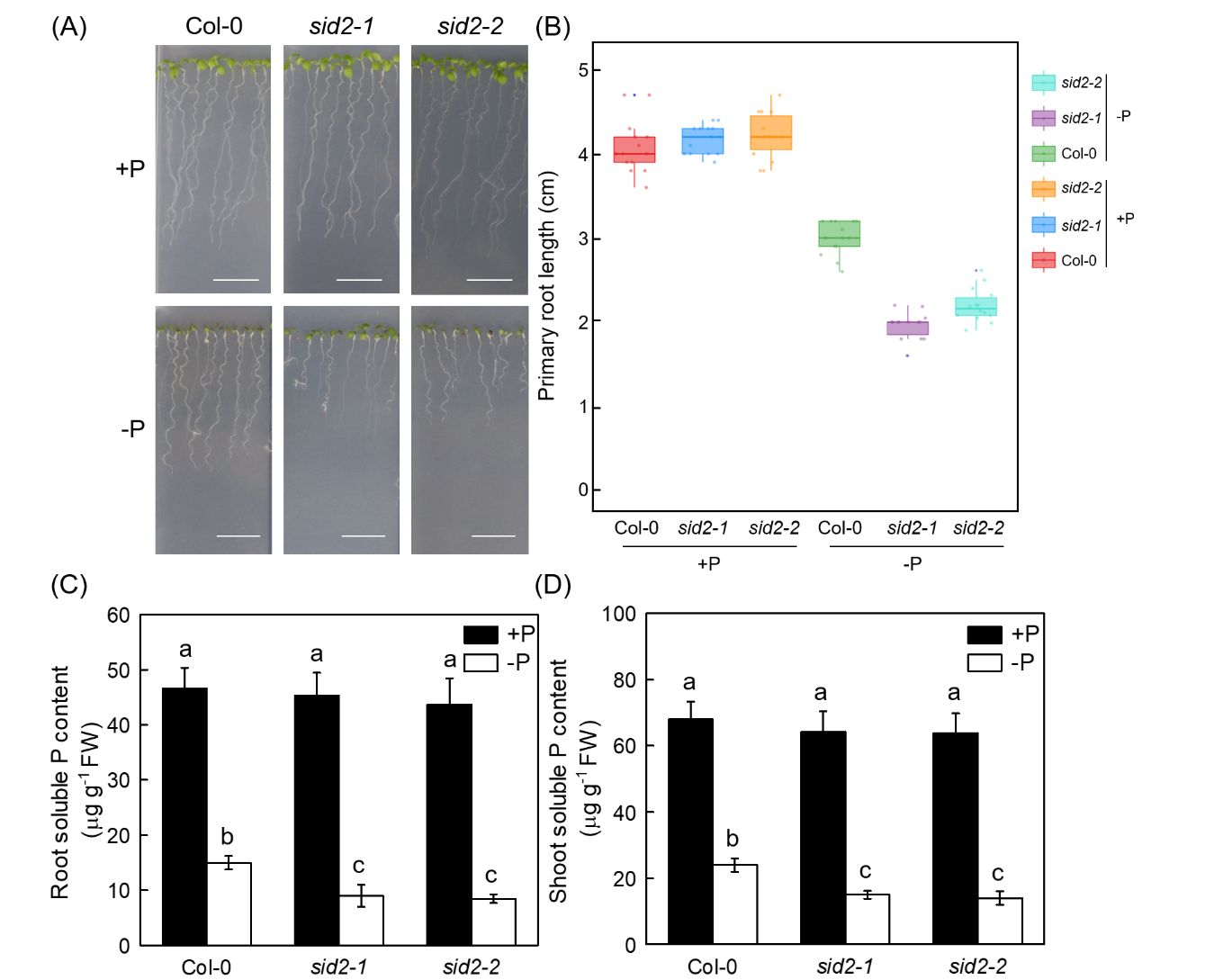


**Supplemental Fig. 2 The phenotype of WT (Col-0) and the *sid2* mutants**

*A. thaliana* seedlings grown in P-deficient (-P) or P-sufficient (+P) conditions for 7 d (A). Primary root length (B), soluble P content in root (C) and shoot (D) were detected. Data are means ± SD (n=4 for soluble P content measurement, n>10 for root length observation). Different letters represent significant differences by Duncan’s multiple range test at the *P*<0.05. Scale bar=1 cm.


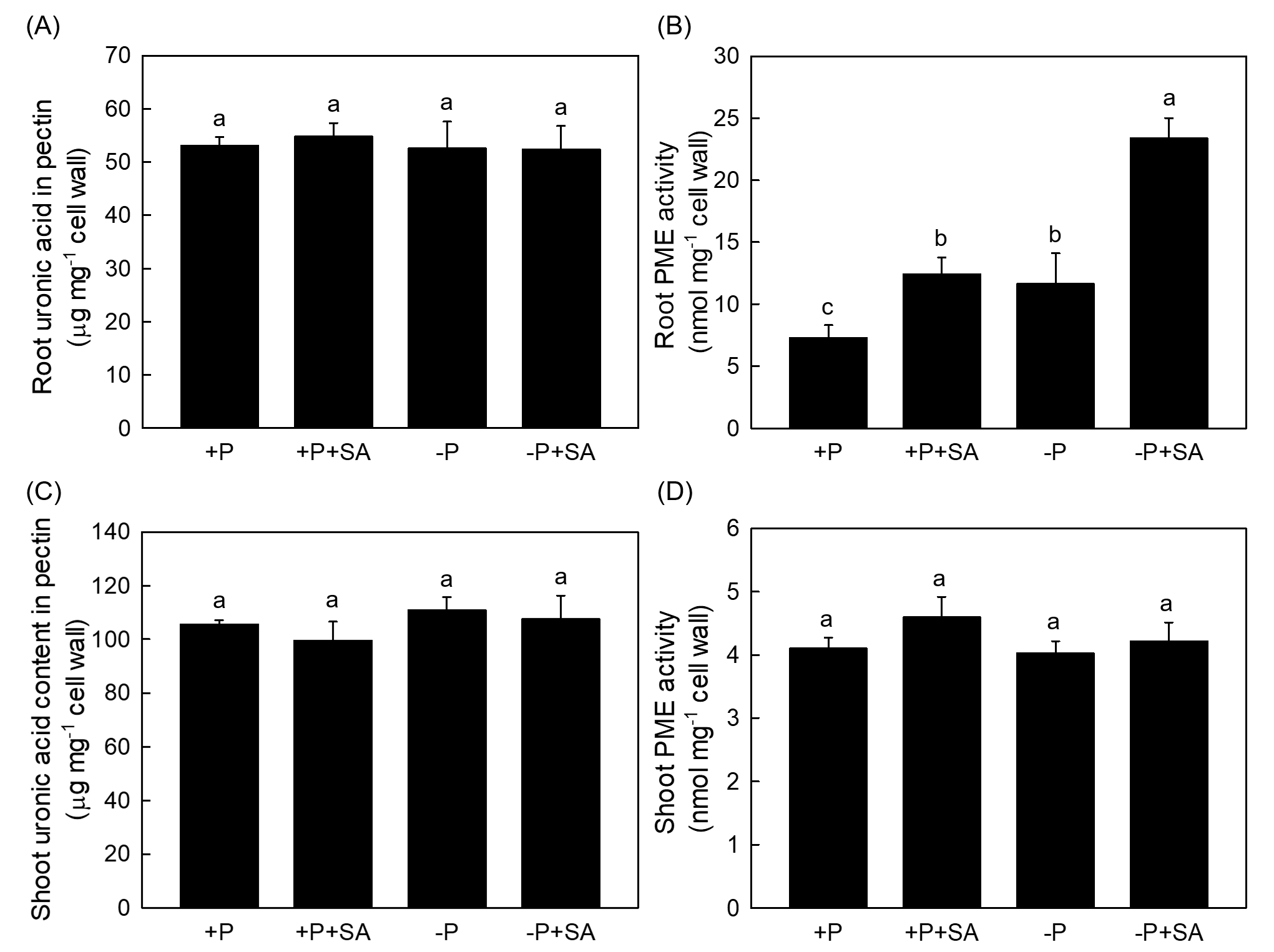


**Supplemental Fig. 3 The effect of SA on cell wall pectin content and PME activity in rice**

Pectin content (A and C) and PME activity (B and D) in root and shoot in the presence or absence of SA under P deficiency were analyzed. Data are means ± SD (n=4). Different letters represent significant differences by Duncan’s multiple range test at the *P*<0.05.


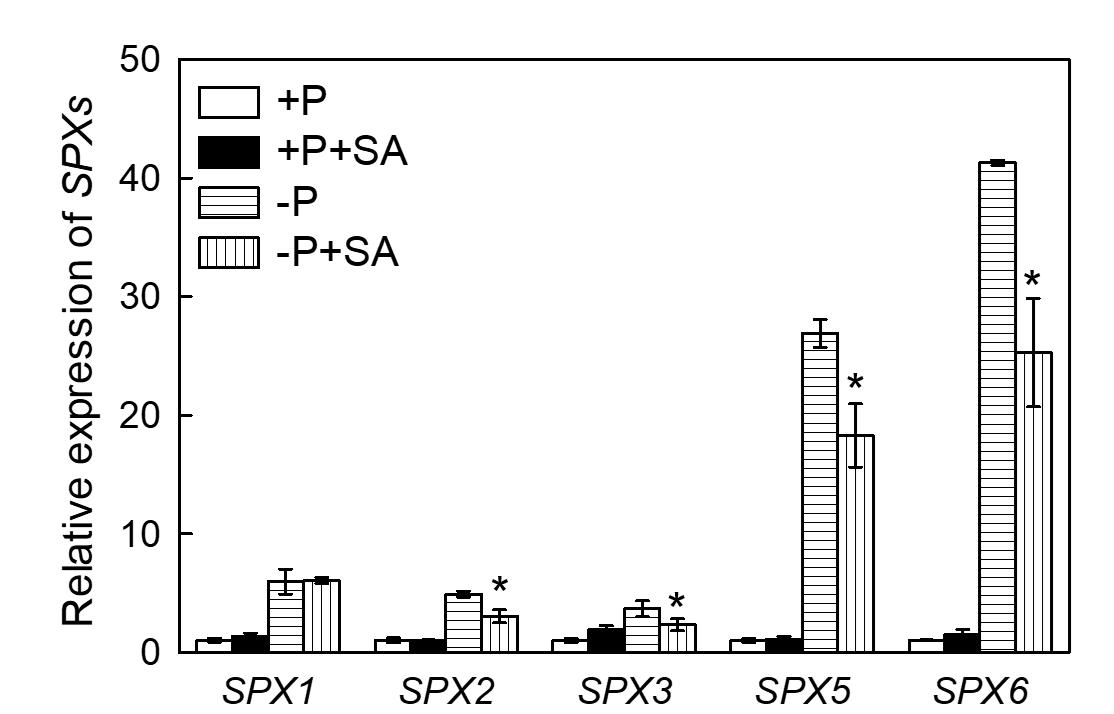


**Supplemental Fig. 4 The effect of SA on P signaling genes in rice under P deficiency**

*SPX1*, *SPX2*, *SPX3*, *SPX5* and *SPX6* mRNA level in roots were detected under –P treatment with or without SA supply were quantitated. Data are means ± SD (n=4) and asterisks indicate a significant difference at *P*<0.05 by Student’s t test.


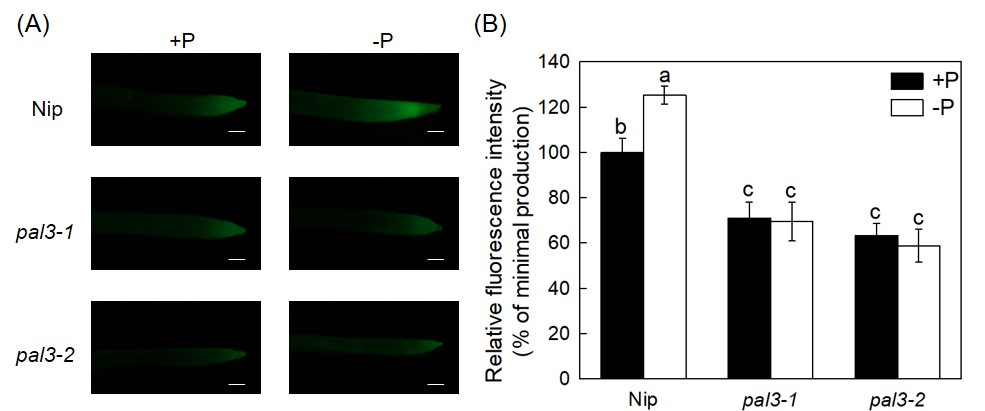


**Supplemental Fig. 5** **The effect of endogenous SA on the NO level in rice**

Root endogenous NO staining (A) indicated by green fluorescence and NO production (B) described as relative fluorescence intensity (% of minimal production) in Nip and *pal3* mutants under respective conditions were displayed (n=8). Different letters represent significant differences by Duncan’s multiple range test at the *P*<0.05. Scale bar = 1 mm.


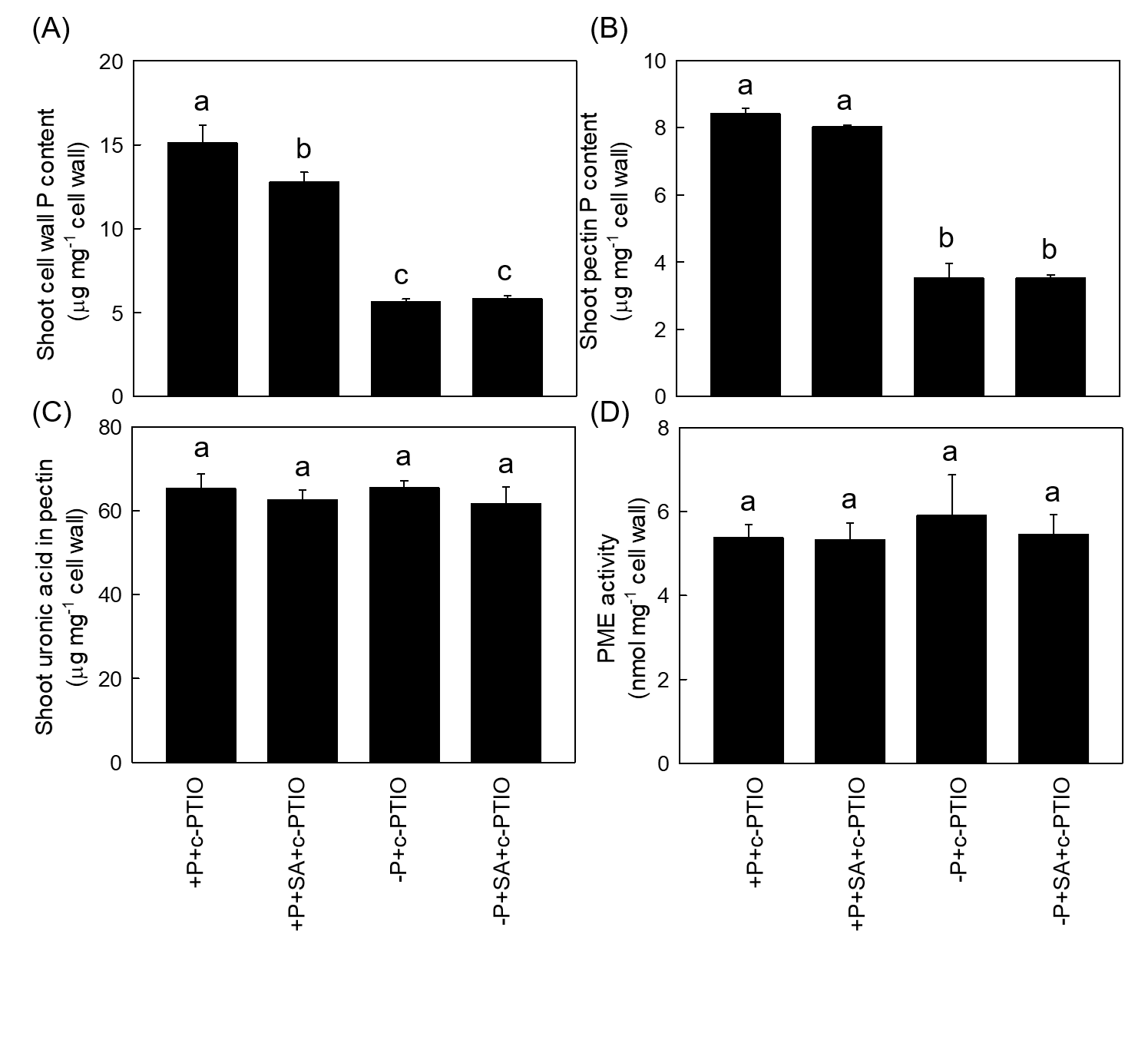


**Supplemental Fig. 6 SA promoted shoot cell wall P reutilization in rice is dependent on NO**

Shoot cell wall P content (A), cell wall pectin P content (B), shoot pectin content (C) and PME activity (D) were measured. Data are means ± SD (n=4). Different letters represent significant differences by Duncan’s multiple range test at the *P*<0.05.


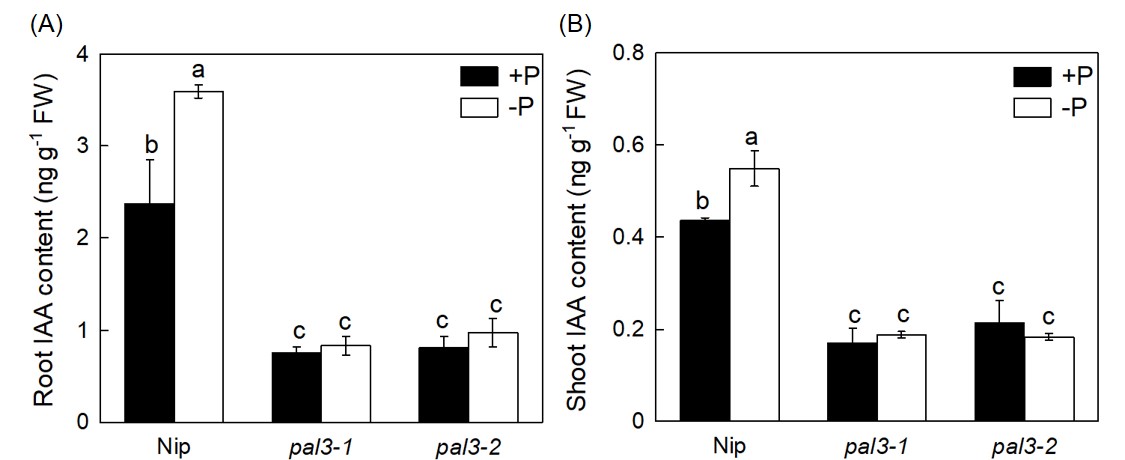


**Supplemental Fig. 7 The effect of endogenous SA on the IAA content in rice**

Root (A) and shoot (B) IAA content in Nip and *pal3* mutants under different conditions were measured. Data are means ± SD (n=4) and different letters represent significant differences by Duncan’s multiple range test at the *P*<0.05.


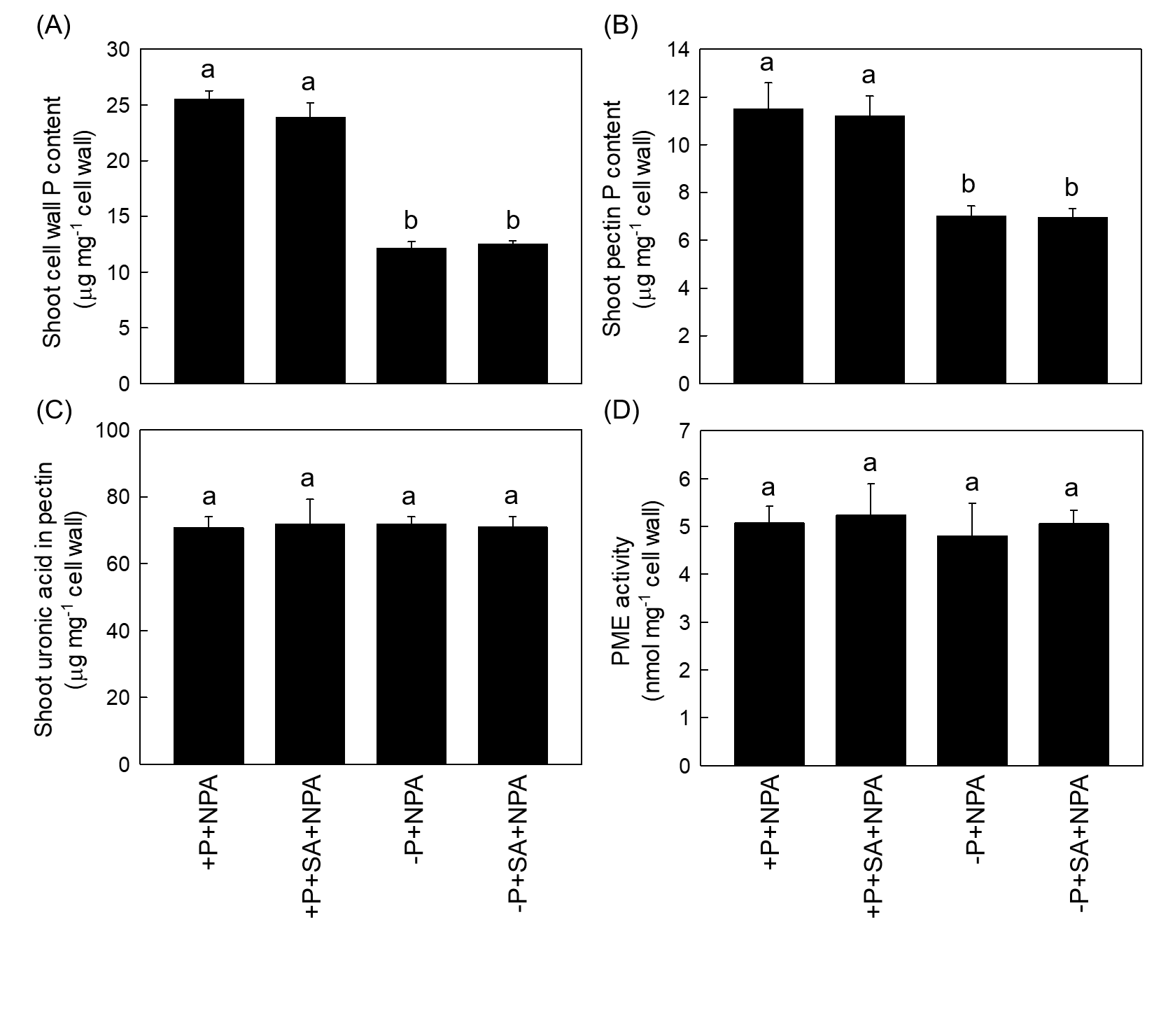


**Supplemental Fig. 8 SA mediated shoot cell wall P reutilization in rice is dependent on auxin**

Shoot cell wall P content (A), cell wall pectin P content (B), shoot pectin content (C) and PME activity (D) were measured. Data are means ± SD (n=4). Different letters represent significant differences by Duncan’s multiple range test at the *P*<0.05.


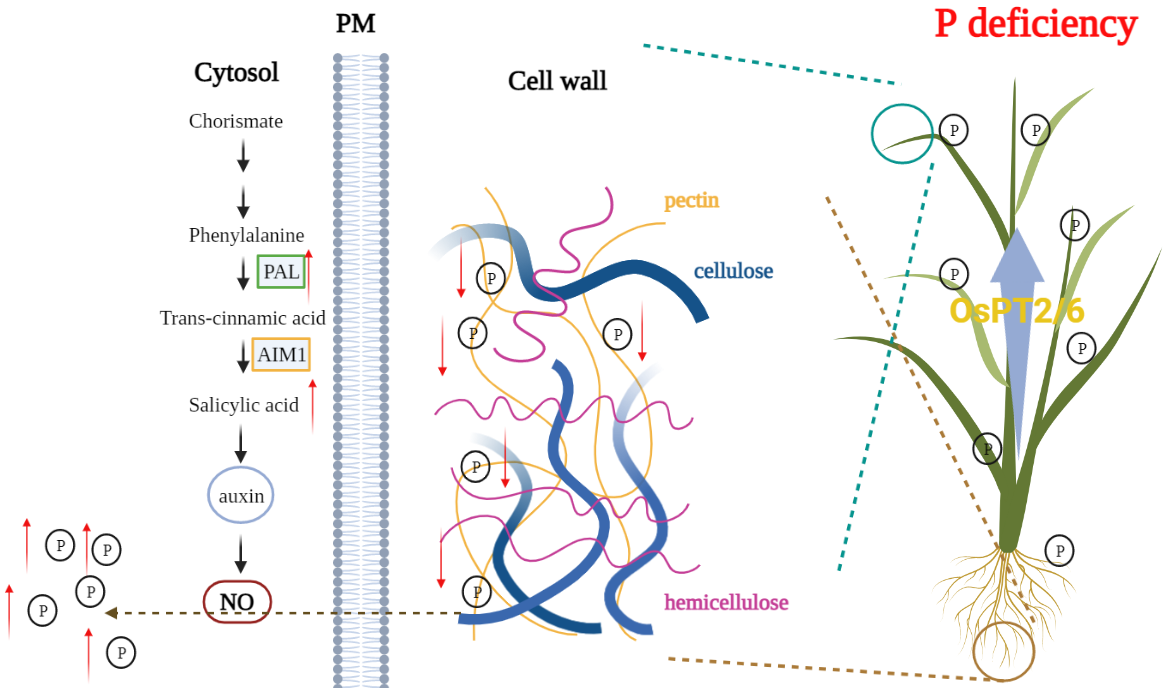

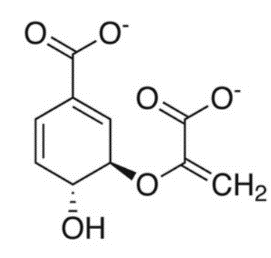

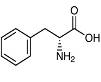

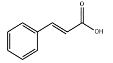

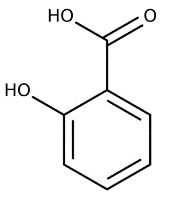


**Supplemental Fig.9 A working model for SA-alleviated P deficiency in rice**

P deficiency induced-SA accumulation could improve the rice growth via two different pathways. One is to activate root and shoot cell wall P remobilization and then solubilize root and shoot cytoplasm P for the survival, which process is dependent on SA-auxin-NO mediated pathway. The other is to facilitate the root-to-shoot P translocation through upregulating the expression of *OsPT2* and *OsPT6.*

**Supplemental Table 1. Primers used in this study**

|  | Forward (5’-3’) | | Reverse (5’-3’) |
| --- | --- | --- | --- |
|  | Primers for qRT-PCR | |  |
| OsActin | TCCGGTGGATCTTCATGCTTACCT | ATGGACCATTGCGACGAGTCTTCT | |
| OsSPX1 | GAAGTTTGGGAAGAGCCTGAGT | TGTAGTTGAGGGCGCTGTAGTT | |
| OsSPX2 | GGAGGTGAAAACGAGAATGG | ACAGCAGGTGGGAAACAAAC | |
| OsSPX3 | TGCAGTCCATCCGATCCG | ATGTGTATGTATGTTCTCTACCACG | |
| OsSPX5 | CGACGAGCTGCAACATT | CAAGAACCATTGGTATTGATC | |
| OsSPX6 | TCTGCGCTGCGAAATCTG | TTGAAAGCCAAAACACGTATG | |
| OsPT2 | GACGAGACCGCCCAAGAAG | TTTTCAGTCACTCACGTCGAGAC | |
| OsPT6 | TATAACTGATCGATCGAGACCAGAG | TGGATAGCCAGGCCAGTTATATATC | |
| OsPT8 | AGAAGGCAAAAGAAATGTGTGTTAAAT | AAAATGTATTCGTGCCAAATTGCT | |
| AIM1 | ATCAATGTGTTCACAGAAGT | TGAATGGCAGCAACTGAAG | |
| PAL1 | AGGAGCTCGGCTGCGTATT | ATGCCGAGGAACACCTTGTT | |
| PAL2 | AGCTGGTCAACGAGTTCTACAACA | GAGGGAGTTGACGTCCTGGTT | |
| PAL3 | AGCACCACCCTGGACAGATC | CTGTCCTGCCTCAGCTTCGT | |
| PAL4 | ATCGACCTGCGCCACATC | GAGTTGGTGCTCAGCGTCTTC | |
| PAL5 | ATCCAAGGTGGCTTCTTCGA | GGCAAGGACAGCAAGAATGTTC | |
| PAL6 | GGGCAACCCAGTGACCAA | CGATTGCCTCGTCGGTCTT | |
| PAL7 | CCAACCCTGTGACCAACCAT | GATCAAGAACGTCGAGGACATG | |
| PAL8 | GCTTCTTCGAGTTGCAGCCTAA | CAGGACCTCGGCGAGGAT | |
| ICS1 | TATGGTGCTATCCGCTTCGAT | CGAGAACCGAGCTCTCTTCAA | |
|  | Primers for CRISPR-Cas9 |  | |
| PAL3-gRNA | CGCTTCTGTCGCGGTCGCTCCTGA | AAACTCAGGAGCGACCGCGACAGA | |
| PAL3-identification | GGGGCACGGTTACCGCGTCC | GAGGCAAGTCCAGAGCCGAC | |
